# 1-Benzyl-2-Phenylbenzimidazole (BPB), a Benzimidazole Derivative, Induces Cell Apoptosis in Human Chondrosarcoma through Intrinsic and Extrinsic Pathways

**DOI:** 10.3390/ijms131216472

**Published:** 2012-12-04

**Authors:** Ju-Fang Liu, Yuan-Li Huang, Wei-Hung Yang, Chih-Shiang Chang, Chih-Hsin Tang

**Affiliations:** 1 Central Laboratory, Shin Kong Wu Ho-Su Memorial Hospital, No.95, Wunchang Road, Shihlin District, Taipei City 111, Taiwan; E-Mail: anti0822@hotmail.com; 2 Graduate Institute of Pharmaceutical Chemistry, China Medical University, No.91 Hsueh-Shih Road, Taichung 40402, Taiwan; E-Mail: chihshiang@mail.cmu.edu.tw; 3 Department of Biotechnology, College of Health Science, Asia University, No.500, Lioufeng Road, Wufeng, Taichung 41354, Taiwan; E-Mail: yuanli@asia.edu.tw; 4 Department of Orthopedic Surgery, Taichung Hospital, Department of Health, No.199, Sec. 1, San-Min Road, Taichung 402, Taiwan; E-Mail: u766018@ms42.hinet.net; 5 Graduate Institute of Biotechnology, National Chung Hsing University, No.250 Kuo Kuang Road, Taichung 402, Taiwan; 6 School of Chinese Medicine, China Medical University, No.91 Hsueh-Shih Road, Taichung 404, Taiwan; 7 Department of Pharmacology, School of Medicine, China Medical University, No.91 Hsueh-Shih Road, Taichung 40402, Taiwan; 8 Graduate Institute of Basic Medical Science, China Medical University, No.91 Hsueh-Shih Road, Taichung 404, Taiwan

**Keywords:** chondrosarcoma, benzimidazole, extrinsic pathway, intrinsic pathway, Chinese herb

## Abstract

In this study, we investigated the anticancer effects of a new benzimidazole derivative, 1-benzyl-2-phenyl -benzimidazole (BPB), in human chondrosarcoma cells. BPB-mediated apoptosis was assessed by the MTT assay and flow cytometry analysis. The *in vivo* efficacy was examined in a JJ012 xenograft model. Here we found that BPB induced apoptosis in human chondrosarcoma cell lines (JJ012 and SW1353) but not in primary chondrocytes. BPB induced upregulation of Bax, Bad and Bak, downregulation of Bcl-2, Bid and Bcl-XL and dysfunction of mitochondria in chondrosarcoma. In addition, BPB also promoted cytosolic releases AIF and Endo G. Furthermore, it triggered extrinsic death receptor-dependent pathway, which was characterized by activating Fas, FADD and caspase-8. Most importantly, animal studies revealed a dramatic 40% reduction in tumor volume after 21 days of treatment. Thus, BPB may be a novel anticancer agent for the treatment of chondrosarcoma.

## 1. Introduction

Chondrosarcomas are malignant tumors showing cartilage differentiation, and it is the third most common primary bone malignancy after myeloma and osteosarcoma. Due to its resistance to both ionizing radiation and chemotherapy, chondrosarcoma is making the management of chondrosarcoma a complicated challenge [1]. Clinically, surgical resection remains the primary mode of therapy for chondrosarcoma. In the absence of an effective adjuvant therapy, this mesenchymal malignancy has a poor prognosis and novel and adequate therapies are needed [2].

Apoptosis is an intracellular suicide program possessing morphologic characteristics and biochemical features, including chromatin condensation, nuclear DNA fragmentation, cell shrinkage, membrane blebbing, and the formation of apoptotic bodies [3,4]. Apoptosis is a physiological mechanism for eliminating malignant cells or cancer cells without eliciting significant damage to normal cells. Thus, induction of apoptosis in target cells is a key mechanism by which anti-cancer therapy works. To date, two major apoptotic pathways have been described as follows: the extrinsic death receptor-mediated pathway and the intrinsic mitochondrion-initiated pathway. The extrinsic apoptotic pathway originates at membrane death receptors (DRs) such as Fas, DR4, and DR5 and then engages the intracellular apoptotic machinery involving adaptor molecules and proximal caspase-8 as well as distal executioner caspases [5]. An apoptotic event engages the intrinsic mitochondrion-dependent processes, affecting mitochondrial permeability and resulting in cytochrome c release, second mitochondria-derived activator of caspase/direct inhibitor of apoptosis-binding protein with low pI (Smac/DIABLO), apoptosis-inducing factor (AIF) and endonuclease G (Endo G) from mitochondria to the cytosol [6]. Cytosolic cytochrome c can trigger the processing of pro-caspase-9 initiating an apoptosome formation composed of Apaf-1, dATP, caspase-9 and cytochrome c [7]. Apoptosome formation leads to the activation of executioner caspase-3, -6 and -7 [8]. Nuclear translocation of AIF and Endo G occurs and can induce DNA fragmentation and apoptotic cell death in a caspase-independent manner [9]. However, it was shown that the release of Endo G and AIF from the mitochondria in response to pro-apoptotic stimuli occurs in a caspase-dependent manner [10].

Benzimidazole derivatives provide useful precursors or subunits for the development molecules of pharmaceutical or biological interest [11]. They have a wide range of biological and pharmacological activities with therapeutic potential. Substituted benzimidazole derivatives have different therapeutic applications, including anti-histamine [12], anti-ulcerative [13], anti-inflammatory [14], anti-oxidant [15], anti-HIV-1 [16], anti-bacterial [17] and anti-cancer activities [18]. We previous reported that benzimidazole derivatives (FPipTB and MPTB) induced cell apoptosis in chondrosarcoma cells [19,20]. In addition, endoplasmic reticulum (ER) stress signaling pathway is involved in benzimidazole derivatives-induced cell death. In this study, we further synthesized a new benzimidazole derivative 1-benzyl-2-phenylbenzimidazole (BPB), and investigated its anticancer activity in human chondrosarcoma cells. Our data provided evidence in human chondrosarcoma cell, that BPB decreased cells survival and tumor growth both *in vitro* and *in vivo*.

## 2. Materials and Methods

### 2.1. Materials

**Figure 1:**
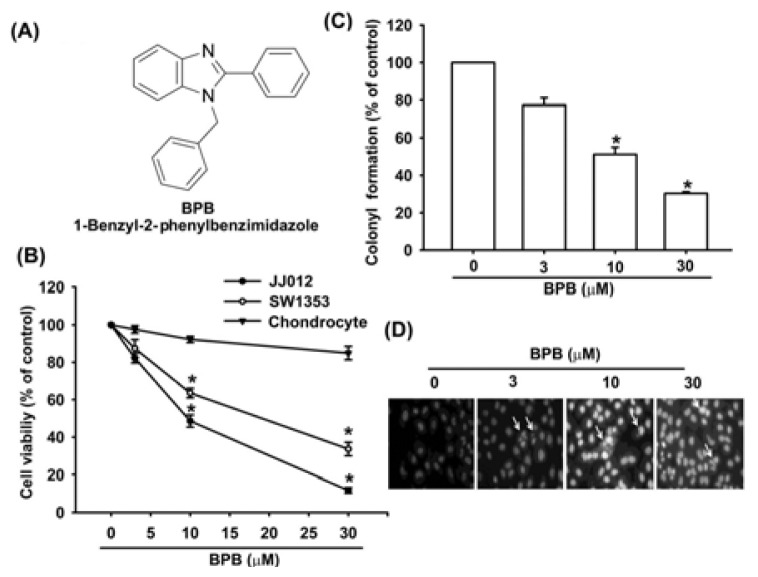
The effect of 1-benzyl-2-phenylbenzimidazole (BPB) on cell viability and colony formation in human chondrosarcoma cells (**A**) Chemical structure of BPB; (**B**) JJ012, SW1353 and chondrocyte cells were incubated with various concentrations of BPB for 48 h, and the cell viability was examined by 3-(4,5-dimethylthiazol-2-yl)-2,5-diphenyltetrazolium bromide (MTT) assay; (**C**) For the colony-forming assay, the clonogenic assay was performed as described in Materials and Methods. The quantitative data are shown; (**D**) JJ012 cells were treated with BPB for 48 h, apoptotic cells were determined by 4′-6-diamidino-2-phenylindole (DAPI) staining and fluorescence microscopy. Results are expressed as the means ± SEM of four independent experiments. ^*^p < 0.05 as compared with control group.

1-benzyl-2-phenylbenzimidazole (BPB: Figure 1A) was synthesized at the Graduate Institute of Pharmaceutical Chemistry, China Medical University (Taichung, Taiwan), and dissolved in DMSO. Horseradish peroxidase-conjugated anti-mouse and anti-rabbit IgG, and rabbit polyclonal antibodies specific for Fas, FADD, cytochrome c, Bcl-2, Bcl-xl, Bax, Bak, Bad, Bid, caspase-8, caspase-3, caspase-9, AIF, Endo G and PARP were purchased from Santa Cruz Biotechnology (Santa Cruz, CA, USA). All other chemicals were obtained from Sigma-Aldrich (St. Louis, MO, USA).

### 2.2. Cell Culture

The human chondrosarcoma cell line JJ012 was kindly provided by Dr. Sean P Scully (University of Miami School of Medicine, Miami, FL, USA) [21]. The human chondrosarcoma cell line SW1353 was obtained from the American Type Culture Collection (Manassas, VA, USA). Cells were cultured in DMEM/α-MEM which were supplemented with 10% fetal bovine serum (FBS) and maintained at 37 °C in a humidified atmosphere of 5% CO_2_.

Primary cultures of human chondrocytes were isolated from articular cartilage as previously described [22,23]. The cells were grown in plastic cell culture dishes in 95% air–5% CO_2_ in DMEM supplied with 20 mM HEPES, 10% heat-inactivated FBS, 2 mM-glutamine, 100 U/mL penicillin and 100 μg/mL streptomycin.

### 2.3. MTT Assay

Cell viability was determined with the 3-(4,5-dimethylthiazol-2-yl)-2,5-diphenyltetrazolium bromide (MTT) assay. After treating with BPB for 2 days, cultures were washed with PBS. Then MTT (0.5 mg/mL) were added to each well and the mixture was incubated at 37 °C for 2 h. To dissolve formazan crystals, culture medium was then replaced with an equal volume of DMSO. After the mixture was shaken at room temperature (RT) for 10 min, absorbance of each well was determined at 550 nm using a microplate reader (Bio-Tek, Winooski, VT, USA) [24].

### 2.4. Colony Assay

To determine the long-term effects of BPB, cells (1000 per well) were treated with BPB at various concentrations for 3 h. After rinsing with fresh medium, cells were allowed to form colonies for 7 days before being stained with crystal violet (0.4 g/L). After washing three times with ddH_2_O, acetic acid was added to a final concentration of 33% (*v*/*v*), and the absorbance was measured at 550 nm.

### 2.5. DAPI Staining

4′-6-diamidino-2-phenylindole (DAPI), a DNA-binding fluorescent dye, was used to determine whether the mechanism of growth inhibition after BPB treatment is through apoptosis. After treatment with BPB for 48 h, the cells were washed three times with PBS, fixed in a 3.7% formaldehyde solution for 10 min, fixed once in 1ml of methanol and then stained with DAPI for 10 min. Results were determined by visual observation of nuclear morphology through fluorescence microscopy.

### 2.6. Quantification of Apoptosis by Flow Cytometry

Apoptosis was assessed by using Annexin V, a protein that binds to phosphatidylserine (PS) residues exposing on the cell surface of apoptotic cells, as previously described [25]. Cells were treated with vehicle or BPB for the indicated times, washed twice with PBS, and resuspended in staining buffer containing 1 μg/mL Propidium iodide (PI) and 0.025 μg/mL Annexin V-FITC. Double-labeling was performed at room temperature for 10 min in the dark, and cells were immediately analyzed by FACScan and the Cellquest program (Becton Dickinson; Lincoln Park, NJ, USA).

Quantitative assessment of apoptotic cells was also assessed by examining the cell cycle. Cells were collected by centrifugation and adjusted to 3 × 10^6^ cells/mL. Pre-chilled ethanol was added to 0.5 mL of cell suspensions and the mixture was incubated at 4 °C for 30 min. Ethanol was then removed by centrifugation, and cellular DNA was stained with 100 μg/mL PI (in PBS containing 0.1% Triton-X 100, and 1 mM EDTA) in the presence of an equal volume of DNase-free RNase (200 μg/mL). After staining, cells were analyzed immediately with a FACScan and Cellquest program. The extent of apoptosis was determined by measuring the DNA content of cells below sub G_1_ peak [26].

### 2.7. Determination of Mitochondrial Membrane Potential

The mitochondrial membrane potential (ΔΨ_m_) was assessed using the fluorometric probe JC-1 (Calbiochem, CA, USA), a positively charged mitochondria-specific fluorophore that indicates depolarization by a fluorescence emission shift from green (525 nm) to red (610 nm) [27]. Briefly, cells were plated in 6-well culture dishes, grown to confluence, and treated with vehicle or BPB. After incubation, cells were stained with JC-1 (5 μg/mL) for 15 min at 37 °C and then analyzed by FACScan using an argon laser (488 nm). Mitochondrial depolarization, which is specifically indicated by a decrease in the red/green fluorescence intensity ratio, was analyzed by Cellquest program.

### 2.8. Western Blot Analysis

Cellular lysates were prepared as previously described [28,29]. Proteins were resolved on SDS-PAGE and transferred to Immobilon polyvinyldifluoride membranes. The blots were blocked with 4% BSA for 1 h at room temperature, and then probed with rabbit anti-human antibodies against Fas, FADD, cytochrome c, Bcl-2, Bcl-xl, Bax, Bak, Bad, Bid, caspase-8, caspase-3, caspase-9, AIF, Endo G or PARP (1:1000 dilution) for 1 h at room temperature. After washed three times, the blots were incubated with a peroxidase-conjugated donkey anti-rabbit secondary antibody (1:1000 dilution) for 1 h at room temperature. The signals were visualized by enhanced chemiluminescence with Kodak X-OMAT LS film (Eastman Kodak, Rochester, NY, USA).

### 2.9. Caspase Activity Assay

The assay is based on the ability of active enzyme to cleave chromophore from enzyme substrate LEHD-pNA (for caspase-9), Ac-IETD-pNA (for caspase-8) or Ac-DEVD-pNA (for caspase-3). Cell lysates were prepared and incubated with anti-caspase-9, -8 and -3. Immunocomplexes were incubated with peptide substrate in assay buffer (100 mM NaCl, 50 mM 4-(2-hydroxyethyl)-1-piperazine-ethanesulphonic acid [HEPES], 10 mM dithiothreitol, 1 mM EDTA, 10% glycerol, 0.1% 3-[(3-cholamidopropyl)dimethylammonio]-1-propanesulfonate [CHAPS], pH 7.4) for 2 h at 37 °C. The release of *p*-nitroaniline was monitored at 405 nm. Results are the percent change in activity compared to untreated control.

### 2.10. siRNA Transfection

siRNA against human AIF, Endo G and control siRNA were purchased from Santa Cruz Biotechnology. Cells were transfected with siRNA (at a final concentration of 2 μg/mL) using Lipofectamine 2000 (Invitrogen Life, Carlsbad, CA, USA) according to the manufacturer’s instructions.

### 2.11. In vivo Tumor Xenograft Study

Male SCID mice (6 weeks old; BALB/cA-nu [nu/nu]) were purchased from the National Science Council Animal Center (Taipei, Taiwan), and maintained in pathogen-free conditions. JJ012 cells (1 × 10^6^ in 200 μL) were injected subcutaneously into the flanks of SCID mice, and tumors were allowed to develop until they reached a size of approximately 100 mm^3^ (~14 days). The mice were treated with vehicle or with 0.5 or 1.5 mg/kg (i.p.; total volume 200 μL) BPB every day for 21 days (10 mice/group). The volume of implanted tumors in the dorsal side of the mice was determined twice a week with a caliper and the formula *V* = *LW*^2^/2, where *V* is volume (mm^3^), *L* is largest diameter (mm), and *W* is smallest diameter (mm). All mice were manipulated in accordance with Animal Care and Use Guidelines of the China Medical University (Taichung, Taiwan) under a protocol approved by the Institutional Animal Care and Use Committee, and conducted in accordance with their guidelines (No.99-5-N; date: 2010/7/3).

To investigate the cell apoptotic effect of BPB in tumor tissues *in vivo*, paraffin-embedded tumor sections were prepared, mounted on slides, deparaffinized in xylene, rehydrated, and washed in distilled water. Protein was removed by digesting the sections with 20 μg/mL proteinase K for 15 min. After washing, labeling was performed by covering the sections with the terminal deoxynucleotidyl transferase-mediated deoxyuridine triphosphate nick end-labeling (TUNEL) reaction mixture at 37 °C for 60 min. The reaction was blocked in stop/wash buffer for 10 min. The TUNEL labeling was visualized using fluorescence microscopy. TUNEL staining was performed using the Apoptosis Detection kit (Trevigen, Gaithersburg, MD, USA).

### 2.12. Statistics

The values reported are means ± SEM. Statistical analysis between two samples was performed using Student’s *t-*test. Statistical comparisons of more than two groups were performed by using one-way analysis of variance (ANOVA) with Bonferroni’s *post-hoc* test. In all cases, *p* < 0.05 was considered significant.

## 3. Results and Discussion

### 3.1. BPB Induces Cell Apoptosis in Human Chondrosarcoma Cells

**Figure 2:**
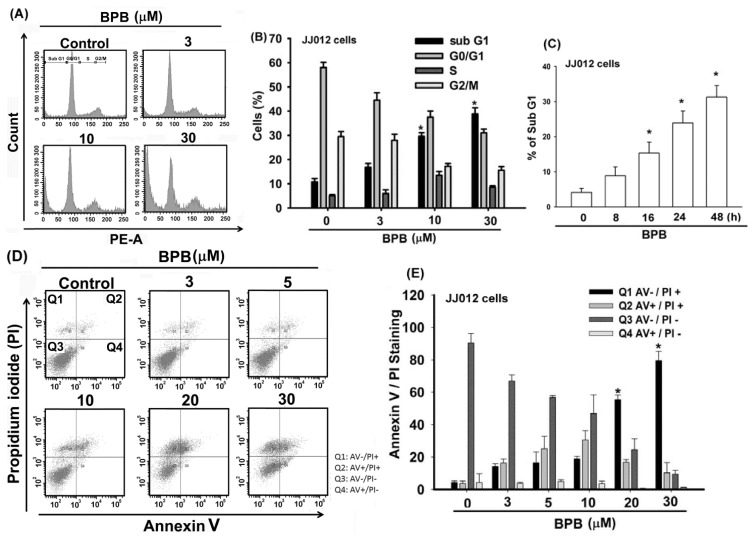

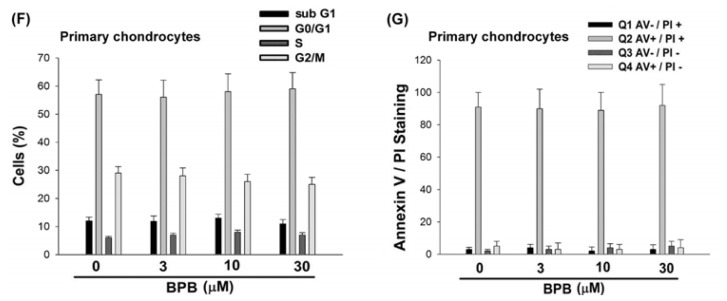
BPB-induced apoptosis of human chondrosarcoma cells. (**A**,**B**,**F**) JJ012 cells or primary chondrocytes were treated with vehicle or BPB for 48 h, and the percentage of apoptotic cells was analyzed by flow cytometry of Propidium iodide (PI)-stained cells. (**C**) JJ012 cells were treated with vehicle or BPB (10 μM) for 48 h, and the percentage of apoptotic cells was analyzed by flow cytometry of PI-stained cells (**D**,**E**,**G**) JJ012 cells or primary chondrocytes were treated with vehicle or BPB for 48 h, and the percentage of apoptotic cells was also analyzed by flow cytometric analysis of Annexin V/PI double staining. Results are expressed as the means ± S.E.M. ^*^p < 0.05 as compared with control group.

To investigate the potential for BPB to induce cell death in human chondrosarcoma cells, we first examined the effect of BPB on cell survival in human chondrosarcoma cells by using the MTT assay. Treatment of cells with BPB induced cell death in chondrosarcoma (JJ012 and SW1353 cells) but not primary chondrocytes (Figure 1B). The IC_50_ values of BPB were 10.7 and 17.5 μM for JJ012 and SW1353 cells, respectively. The anti-cancer activities of BPB were further assessed with clonogenic assays, which correlated very well with previous *in vivo* assays of tumorigenicity in nude mice [30]. Treatment of JJ012 cells with BPB reduced colony formation dose-dependently (Figure 1C). We next investigated whether BPB induces cell death through an apoptotic mechanism by DAPI staining, PI and Annexin V/PI assay. Treatment of JJ012 cells with BPB significantly increased the condensation of chromatin by DAPI staining using immunofluorescence microscopy (Figure 1D). In addition, treating cells with BPB induced a concentration- and time- dependent increase in cell death, resulting in an increase in the percentage of cells in the sub G1 phase (Figure 2A–C). Annexin V/PI double-labeling was used to detect PS externalization, a hallmark of the early phase of apoptosis. Compared to vehicle-treated cells, a high proportion of annexin V labeling was detected in cells treated with BPB (Figure 2D,E). On the other hand, BPB also did not increase cell apoptosis in primary chondrocytes by PI and Annexin V staining (Figure 2F,G)

**Figure 3:**
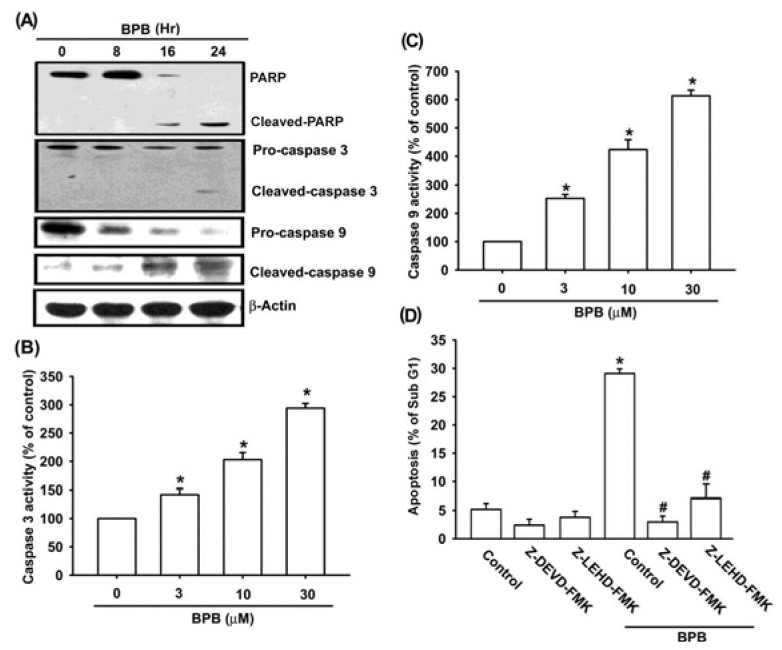
BPB induced the activation of caspases in human chondrosarcoma cells. (**A**) JJ012 cells were incubated with BPB (10 μM) for different time intervals, and the PARP, caspase-3 and caspase-9 expression were examined by Western blot analysis; (**B**,**C**) JJ012 cells were incubated with BPB for 24 h, and then caspase-3 and caspase-9 activities were examined by caspase ELISA kit; (**D**) Cells were pre-treated for 30 min with z-DEVD-FMK (caspase 3 inhibitor) or z-LEHD-FMK (caspase 9 inhibitor), and then followed by stimulation with BPB for 48 h, and the percentage of apoptotic cells was analyzed by flow cytometry of PI-stained cells. Results are expressed as the means ± SEM. ^*^p < 0.05 as compared with control group. # *p* < 0.05 compared with BPB-treated group.

One of the hallmarks of the apoptotic process is the activation of cysteine proteases, which include both initiators and executors of cell death. Treatment with BPB increased expression of cleaved caspase-8 and related caspase activation (Figure 3A,C). BPB also increased the expression of cleaved caspase-8 and related activation (Figure 3A,B). Pretreatment of cells with the specific caspase-3 inhibitor (z-DEVD-FMK) or the specific caspase-9 inhibitor (z-LEHD-FMK) reduced BPB-induced cell death, as shown by PI-staining (Figure 3D). On the other hand, BPB also increased cleaved-PARP (Figure 3A). These data indicate that BPB induced cell death through an apoptosis mechanism

### 3.2. Intrinsic and Extrinsic Pathways Are Mediates BPB-Induced Cell Apoptosis in Human Chondrosarcoma Cells

**Figure 4:**
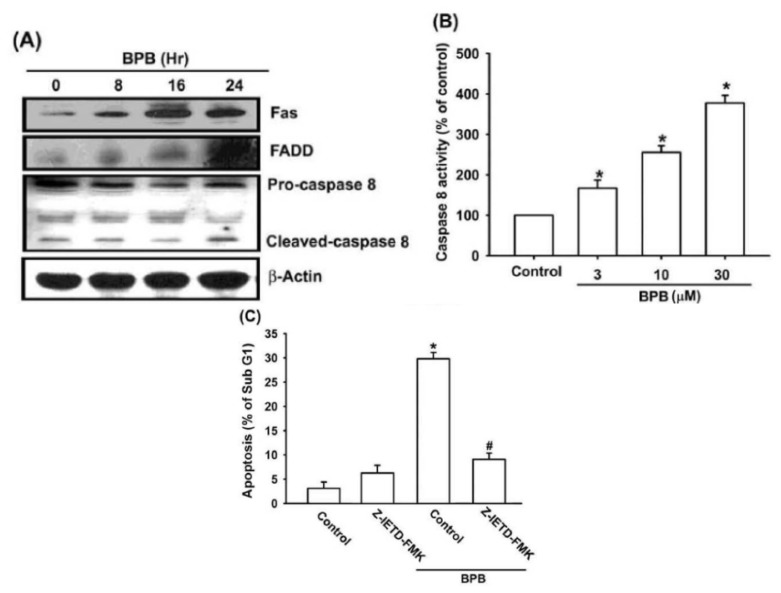

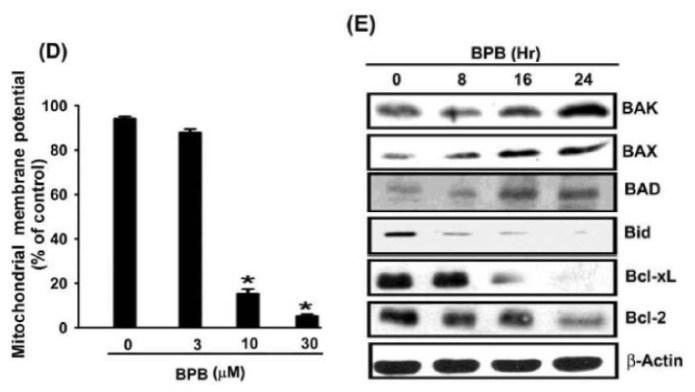
Intrinsic and extrinsic pathways are involved in BPB-induced cell apoptosis in human chondrosarcoma cells. (**A**) JJ012 cells were incubated with BPB (10 μM) for different time intervals, and Fas, Fas-associated protein with death domain (FADD) and caspase-8 expression were examined by Western blotting; (**B**) JJ012 cells were incubated with BPB for 24 h, and then caspase-8 activation was examined by caspase-8 ELISA kit; (**C**) Cells were pretreated for 30 min with z-IED-FMK (caspase-8 inhibitor) followed by stimulation with BPB (10 μM) for 48 h, and the percentage of apoptotic cells was determined by flow cytometric analysis of cell cycle; (**D**) JJ012 cells were incubated with various concentration of BPB for 48 h, and the mitochondrial membrane potential was examined by flow cytometry; (**E**) JJ012 cells were incubated with BPB (10 μM) for different time intervals, and Bax, Bak, Bad, Bcl-2 and Bcl-xl expression were examined by Western blotting. Results are expressed as the means ± SEM. ^*^p < 0.05 compared with control group. # *p* < 0.05 compared with BPB-treated group.

It is well-known that apoptosis can be activated through two main pathways: the intrinsic mitochondria-dependent pathway and the extrinsic death receptor-dependent pathway [31]. Fas, Fas-associated protein with death domain (FADD) and caspase-8 play important roles in death receptor-dependent pathway of apoptosis [32]. We examined whether BPB induced apoptosis by triggering the extrinsic apoptotic pathway. As shown in Figure 4A, BPB induced an increase in Fas and FADD protein levels. Treatment of cells with BPB also increased expression of cleaved caspase-8 and related caspase activation (Figure 4A,B). In addition, pretreatment of cells with the specific caspase-8 inhibitor (z-IED-FMK) reduced BPB-induced cell death in chondrosarcoma (Figure 4C). Therefore, extrinsic death receptor-dependent pathway is involved in BPB-induced cell apoptosis in human chondrosarcoma cells.

To explore whether BPB-induced apoptosis is mediated by mitochondrial dysfunction, we determined the mitochondrial membrane potential with the mitochondria-sensitive dye JC-1 using flow cytometry. As shown in Figure 4D, treatment of JJ012 cells with BPB for 48 h induced the loss of the mitochondrial membrane potential in a dose-dependent manner. On the other hand, treatment of JJ012 cells with BPB induced Bad, Bax and Bak protein levels (Figure 4E). In addition, BPB decreased the expression of Bid, Bcl-XL and Bcl-2, which led to an increase in the proapoptotic/antiapoptotic Bcl-2 ratio (Figure 4E). These data suggest that BPB-increased cell death is mediated by mitochondrial dysfunction in human chondrosarcoma cells.

### 3.3. BPB Increases Cytochrome c, AIF, and Endo G Release in Chondrosarcoma Cells

**Figure 5:**
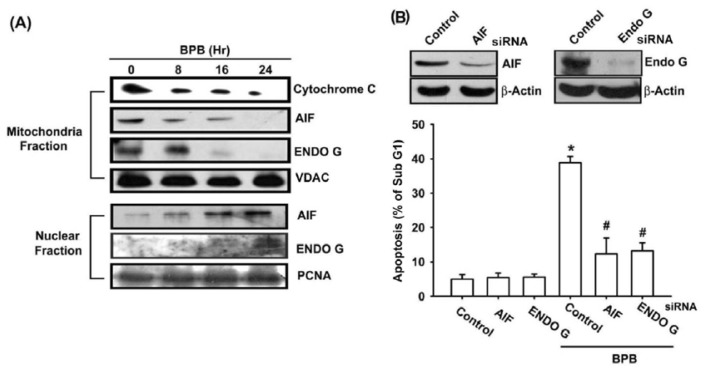
Apoptosis-inducing factor (AIF) and endonuclease G (Endo G) are involved in BPB-induced cell death. (**A**) JJ012 cells were incubated with BPB (10 μM) for different time intervals, and the levels of cytochrome c, AIF and Endo G in mitochondria and nuclei were examined by Western blot analysis. (**B**) Cells were transfected with AIF, Endo G or control siRNA for 24 h, and the AIF and Endo G expression were examined by Western blot analysis (upper panel). Cells were transfected with AIF, Endo G or control siRNA for 24 h, and then followed by stimulation with BPB (10 μM) for 48 h, and the percentage of apoptotic cells was analyzed by flow cytometry of PI-stained cells (lower panel). Results are expressed as the means ± SEM. ^*^p < 0.05 compared with control; # *p* < 0.05 compared with BPB-treated group.

The release of pro-apoptotic proteins (e.g., cytochrome c, AIF, and Endo G) from the mitochondrial to cytoplasm is a critical event that occurs during mitochondrial-dependent pathway [33]. BPB significantly decreased the mitochondrial cytochrome c, AIF, and Endo G as compared with the control group (Figure 5A). We next explored whether BPB increased the release of the AIF and Endo G from the mitochondria into the nuclei. Immunoblot data showed that although control cells demonstrated a lack of nuclear expression of AIF and Endo G, BPB treatment induced demonstrable translocation of AIF and Endo G to the nuclei (Figure 5A). To further investigation whether BPB induced apoptosis through AIF and Endo G, we then transfected AIF and Endo G siRNA to JJ012 cells for 24 h. Our results indicate that siRNA transfection inhibited the expression of AIF and Endo G, and blocked BPB-induced cell death (Figure 5B). These observations demonstrate that BPB induces apoptosis through AIF and Endo G translocation to the nuclei in human chondrosarcoma cells.

### 3.4. BPB Inhibits Tumor Growth in the Mouse Xenograft Model of JJ012 Cells

**Figure 6:**
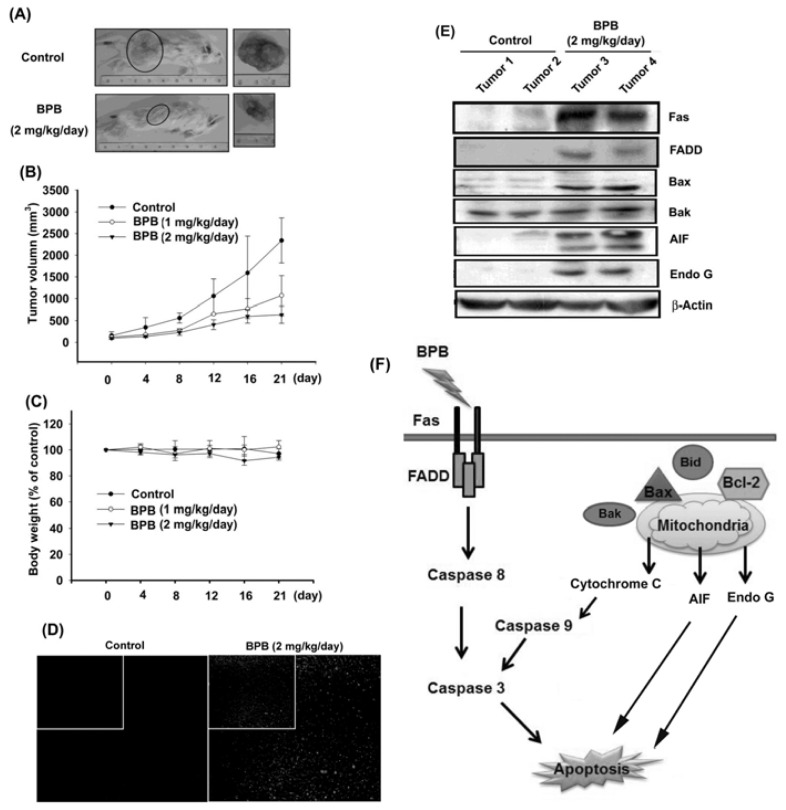
Effects of BPB on tumorigenicity and *in vivo* growth of xenografts in SCID mice. (**A**,**B**) The chondrosacoma cells (1 × 10^6^) were injected subcutaneously into the 5-week-old SCID mice. After the tumors reached 100 mm^3^ in size, the animals were treated with an intraperitoneal injection of BPB (1 or 2 mg/kg) or vehicle daily for 3 weeks. The mean tumor volume was measured at the indicated number of days after implantation (*n* = 8–10). (**C**) Mean body weight was measured at the indicated number of days after implantation. (**D**) Transferase-mediated deoxyuridine triphosphate nick end-labeling (TUNEL) assay in tissues from chondrosarcoma cells xenografts. BPB-treated tumors show marked green staining of fragmented nuclei, indicative of apoptosis. (**E**) The expression of Fas, FADD, Bax, Bak, AIF and Endo G were evaluated by Western blot analysis in tumor with and without treatment. (**F**) The proposed signaling pathways of BPB-induced cell apoptosis in human chondrosarcoma cells.

On the basis of the BPB-induced apoptotic effect exhibited *in vitro*, we decided to detect whether BPB possessed antitumor activities *in vivo*. Therefore, we established xenografts of JJ012 cells in SCID mice; as tumors reached 100 mm^3^ in size, the mice were divided into three groups and treated with either vehicle or BPB. BPB induced a dose-dependent inhibition of tumor growth (Figure 6A,B). Moreover, in these two animal models, body weights were not significantly affected by BPB (Figure 6C). On the other hand, an increase of TUNEL-positive cells was observed in tumors of the BPB-treated mice when compared with tumors taken from vehicle-treated mice (Figure 6D). Finally, *ex vivo* analysis of tumors excised from mice showed significantly increasing Fas, FADD, Bax, Bak, AIF and Endo G expression in the BPB-treated group compared with that in the control group, as shown by Western blot (Figure 6E). Taken together, these results suggest that BPB inhibits tumor growth by inducing JJ012 cell apoptosis *in vivo*.

## 4. Discussion and Conclusions

It is documented that osteosarcoma and Ewing’s sarcoma are dramatic increase in long-term survival with the advent of systemic chemotherapy. However, chondrosarcoma continue to have a poor prognosis due to absence of an effective adjuvant therapy [34]. The development of novel therapeutic agents targeting the malignant behavior of chondrosarcoma cells is important to improve the prognosis. Benzimidazole derivatives have been demonstrated to possess the effects of anti-bacterial, anti-fungal, and anti-viral [16,17,35]. It also has been reported that benzimidazole derivatives induced anti-mitotic and anti-cancer effects in many human cancer cells [36]. We previously reported that benzimidazole derivatives (FPipTB and MPTB) induced cell apoptosis in human chondrosarcoma cells [19,20]. In addition, both FPipTB and MPTB induced cell death through ER stress signaling pathway. Here, we synthesized a new benzimidazole derivative 1-benzyl-2-phenylbenzimidazole (BPB) and examined its anticancer effect in human chondrosarcoma cells. We found that BPB induced cell death in human chondrosarcoma cell lines but not primary chondrocytes. Furthermore, we also showed that the intrinsic and extrinsic signaling pathways are involved in BPB-mediated cell death. In contrast, treatment of cells with BPB also increased ER stress and calcium/calpain activation (data not shown), suggesting that ER stress involved in BPB-mediated cell apoptosis. We next hypothesized that Endo G and AIF may participate in FPipTB and MPTB-induced cell death. Indeed, treating cells with FPipTB and MPTB enhanced Endo G and AIF activation (data not shown). Therefore, ER stress activation as well as intrinsic and extrinsic signaling pathways are common pathways in benzimidazole derivatives-mediated cell death in human chondrosarcoma.

The process of apoptosis is controlled by two diverse cell signals, which can be initiated by two major pathways: intrinsic and extrinsic pathway [32]. The extrinsic pathway induces activation of caspase-8, caspase-10 and caspase-3 through Fas receptor [37]. The interaction between Fas and FasL results in the formation of the death-inducing signaling complex (DISC), which contains the FADD, caspase-8 and caspase-10. Here, we found that BPB increased Fas and FADD expression. In addition, BPB also increased the expression and activity of caspase-8. Pretreatment of cells with caspase-8 inhibitor reduced BPB-induced cell death. These data suggests that extrinsic death-receptor pathway is involved in BPB-induced cell apoptosis.

Mitochondrial dysfunction has been implicated as being a key mechanism in apoptosis in various cell death paradigms [38]. Two major events have been noted in apoptosis involving mitochondrial dysfunction. One event is the change in the membrane permeability and subsequent loss of membrane potential [39]. The other is the release of apoptotic proteins including cytochrome c, AIF and Endo G from the inter-membrane space of mitochondria into the cytosol [40]. Here, we also found that BPB reduced mitochondria membrane potential and increased the release of cytochrome c, AIF and Endo G. Bcl-2 family proteins regulate mitochondria-dependent apoptosis with the balance of anti- and pro-apoptotic members arbitrating life-and-death decisions [41]. On the other hand, BPB treatment results in a significant increase of Bax, Bad, and Bak expression, and decrease of Bcl-2, Bcl-XL, and Bid, suggesting that changes in the ratio of pro-apoptotic and anti-apoptotic Bcl-2 family proteins might contribute to apoptosis-promotion activity of BPB. In agreement of these observations, we noted that the mitochondrial dysfunction may be involved in BPB-induced cell apoptosis of human chondrosarcoma cells.

Caspase-independent apoptotic pathways are important safeguard mechanisms to protect organisms against unwanted and potentially cancer cells [42]. AIF and Endo G released from mitochondria are important event in caspase-independent apoptotic pathways [43]. Our results show that BPB induced AIF and Endo G translocation from mitochondria to nucleus, and suggests that BPB-induced apoptosis also involves caspase-independent mechanisms. Besides, transfection of cells with AIF or Endo G siRNA remarkably attenuated BPB-induced apoptosis, demonstrating the dominant roles of AIF and Endo G in BPB-induced apoptosis in human chondrosarcoma cells. These data suggest that caspase-independent pathway is also involved in BPB-induced cell death by apoptosis.

In conclusion, our data indicate that the novel benzimidazole derivative BPB induces cell death in human chondrosarcoma cells both *in vitro* and *in vivo*. We propose the signaling pathway of BPB induced apoptosis in chondrosarcoma cells as shown in Figure 6F. First, the extrinsic pathway is initiated by ligation of transmembrane death receptor (Fas and FADD) to activate membrane-proximal caspases (caspases-8), which in turn cleave and activate effector caspases such as caspases-3. Second, the intrinsic pathway requires disruption of the mitochondrial membrane and the release of mitochondrial proteins, such as cytochrome c, which is released from the mitochondrial intermembrane space to cytoplasm, which in return induces activation of caspase-9 and thereby initiates the apoptotic caspase cascade. In addition, AIF and Endo G also translocates from mitochondrial to nucleus during mitochondrial dysfuncton to execute capsase-independent apoptosis. Thus, BPB is a promising chemotherapeutic agent worthy of further development for treatment of human chondrosarcoma cells.
